# ER81 Expression in Breast Cancers and Hyperplasia

**DOI:** 10.4061/2011/980513

**Published:** 2011-02-27

**Authors:** YuanYuan Wang, Li Wang, Yue Chen, Lin Li, XuanTao Yang, BaoLin Li, ShuLing Song, LiLin Yang, Yan Hao, JuLun Yang

**Affiliations:** ^1^Department of Pathology, Kunming General Hospital/Kunming Medical College, Kunming, Yunnan 650032, China; ^2^Department of Pathology, The First People's Hospital of Yunnan Province, Kunming, Yunnan 650031, China; ^3^Neuroscience Division, Lilly Corporate Center, Eli Lilly and Company, Indianapolis, IN 46285, USA

## Abstract

ER81 is a transcription factor that may contribute to breast cancer; however, little known about the role of ER81 in breast carcinogenesis. To investigate the role of ER81 in breast carcinogenesis, we examined ER81 expression in IDC, DCIS, ADH, HUT, and normal breast tissues by immunohistochemical staining. We found that ER81 overexpression was detected in 25.7% (9/35) of HUT, 41.2% (7/17) of ADH, 54.5% (12/22) of DCIS, and 63.0% (51/81) of IDC. In 20 of breast cancer tissues combined with DCIS, ADH, and HUT, ER81 expression was found in 14/20 (70%) IDC. In these 14 cases all cases were ER81 positive expression in DCIS, 13 of 14 cases were positively expressed of ER81 in ADH and 8 of 14 were positive for ER81 in HUT components. A statistical significance was found between NBT and HUT (*P* < .05) and HUT and ADH (*P* < .05). Clinical-pathological features analysis of breast cancer revealed that ER81 expression was significantly associated with Her2 amplification and was negatively associated with ER and PR expression. Our results demonstrated that ER81 overexpression was present in the early stage of breast development that suggested that ER81 overexpression may play an important role in breast carcinogenesis.

## 1. Introduction

Breast carcinogenesis is thought to undergo a transition from normal epithelium to invasive carcinoma (IDC) via hyperplasia of usual type (HUT), atypical ductal hyperplasia (ADH), and carcinoma in situ (DCIS) [1]. Over 14% of breast cancer diagnosed in the United States annually are DCIS, and approximately 50% of untreated DCIS will develop into an IDC within 24 years after the original biopsy [2]. However, it is unclear how IDC develop from these lesions. 

ER81 (ETS-related 81), also called ETS variant 1 (ETV1), is a transcription factor that is a member of the ETS family of DNA-binding proteins [3–5]. Its association with cancer was first noted in Ewing tumors, in which the EWS gene can be translocated onto the ER81 gene and the resultant EWS-ER81 fusion protein exerts oncogenic properties [6]. From then on, many findings suggest that dysregulation of ER81 target genes in disparate tumors like Ewing sarcomas and prostate carcinomas are causally involved in tumorigenesis [7]. Of note, ER81 transcriptional activity is dramatically enhanced upon Her2/Neu (a receptor tyrosine kinase and proto-oncoprotein especially associated with breast cancer) overexpression [8, 9]. Moreover, ER81 mRNA levels are increased in murine cell lines and tumors overexpressing Her2/Neu and also in many human breast cancer cell lines, which suggests that ER81 may contribute to breast tumorigenesis [10–12]. 

To gain more insight into ER81's role in breast tumorigenesis, we attempted to examine ER81 expression in invasive breast cancers, ductal carcinoma in situ, atypical ductal hyperplasia, and hyperplasia without atypia and normal breast tissues in this study.

## 2. Materials and Methods

### 2.1. Materials

Formalin-fixed, paraffin-embedded tissue specimens including 103 of primary breast cancers including 81 invasive ductal carcinomas (IDC) and 22 ductal carcinomas in situ (DCIS), 52 of breast hyperplasia including 17 atypical ductal hyperplasia (ADH) and 35 hyperplasia of usual type (HUT), and 62 of normal breast tissues (NBT) were collected from Department of Pathology, Kunming General Hospital and The First People's Hospital of Yunnan Province between June 2006 and October 2009. Furthermore, 20 paraffin-embedded tissue blocks of breast invasive ductal carcinomas combined with ductal carcinomas in situ, atypical Hyperplasia, hyperplasia of usual type, and normal breast tissues were got from the two hospitals.

### 2.2. Immunohistochemical Staining

Immunohistochemistry was employed to detect the expression of ER81 for all breast tissues and the expression of ER, PR for all the 81 cases of invasive ductal carcinomas tissues. EnVision Systems was adopted for the staining. Briefly, 4 *μ*m sections were taken from formalin-fixed, paraffin-embedded tissue blocks. The deparaffinized sections were pretreated with heat-induced epitope retrieval and then treated with 30 mL/L hydrogen peroxidase in methanol for 30 min to block endogenous peroxidase activity. The sections were further blocked with 10 mL/L normal goat serum for 30 min, followed by incubation with primary antibody (mouse antihuman monoclonal antibody ER81, SANTA CRUZ BIOTECHNOLOGY, INC; mouse antihuman monoclonal antibody ER and PR, Maixin-Bio, Fuzhou, China) at 4°C overnight. The sections were then washed in 0.01 mol/L phosphate buffer solutions (PBS, pH 7.2) and sequentially incubated with Envision (Envision kit, DakoCytomation, Inc, Carpinteria, California, USA) for 30 min. The reaction product was visualized by diaminobenzidine tetrahydrochloride (DAB). All slides were counterstained with hematoxylin, dehydrated, and mounted. PBS substituting for the primary antibody was used as the negative control.

### 2.3. Assessment of Immunohistochemical Staining

Specific staining was evaluated independently by two investigators. We used a semiquantitative manner to assess the ER81 staining, yielding an immunoreactive score (IRS) ranging from 0 to 9. IRS was calculated by multiplying the number of positive cytoplasmic staining of cells (0  =  none, 1 = <10%, 2 = 10–50%, 3 = >50% positive tumor cells) by the staining intensity (1  =  weak, 2  =  moderate, 3  =  strong). Then we considered IRS 0 score as ER81 expression “−”, IRS 1-2 score as ER81 expression “+”, IRS 3–5 score as ER81 expression “++”, IRS 6–9 score as ER81 expression “+++”. Positive reaction in a normal epithelium yielded a maximum IRS of 2. Therefore breast hyperplastic cells and breast cancer cells in other groups were considered ER81 positive with IRS ≥ 3 as suggested by Going et al. [13]. The ER and PR positive staining should be localized to the nucleus. Specimens in which more than 10% of cells showed positive immunoreactivity were considered to be immunoreactive.

### 2.4. Fluorescence In Situ Hybridization (FISH)

A HER2/neu probe kit (China Medical Technologies, Inc, Beijing, China) was used for FISH analysis for all the 81 cases of invasive ductal carcinomas tissues. Tissue sections were baked overnight at 56°C, dewaxed in xylene, dehydrated and air-dried. The slides were then pretreated with sodium bisulfite at 50°C for 30 min and digested with protease K for 15 min at 37°C and finally hybridized overnight at 42°C with the probes (GLP HER2/CSP17 DNA probe, China Medical Technologies, Inc, Beijing, China) after DNA denaturation at 73°C. Slides were washed with posthybridization buffer at 73°C, counterstained with 4, 6-diamidino-2-phenylindole (DAPI) and mounted and stored in the dark prior to signal enumeration. For FISH analysis, slides were examined with fluorescence microscope. Areas of optimal tissue digestion and no overlapping nuclei were then selected in each core for counting. 30 cells were counted for each case. We considered cases with a FISH ratio (Her2 gene signals to chromosome 17 signals) of ≥2.2 as Her2 amplified.

### 2.5. Statistical Analysis

The statistical analysis was performed using the SPSS software package, version 11.0. The differences were analyzed by Kruskal-Wallis (K-W or H) test and Pearson chi-squared distribution (*χ* ^2^) test. A value of *P* < .05 was considered statistically significant.

## 3. Results

**Figure 1:**
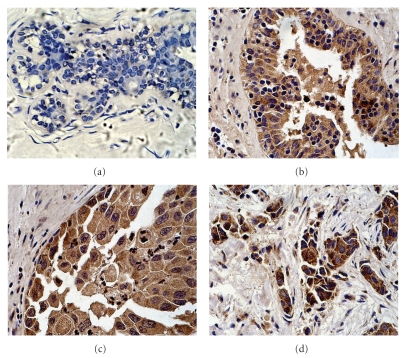
ER81 expression in breast cancers and hyperplasia. (a) Negative expression of ER81 in hyperplasia of usual type (×400). (b) Positive expression of ER81 in atypical ductal hyperplasia (×400). (c) Positive expression of ER81 in ductal carcinoma in situ (×400). (d) Positive expression of ER81 in invasive ductal carcinoma (×400).

**Figure 2:**
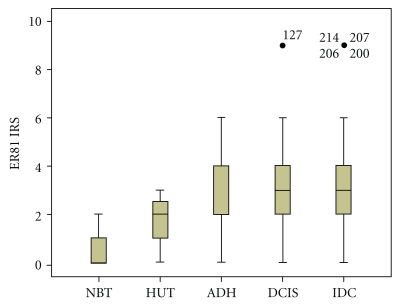
Comparison of ER81 expression in breast cancers and hyperplasia. The box plot markings represent median, 25–75th percentile, and the range of all values.

**Figure 3:**
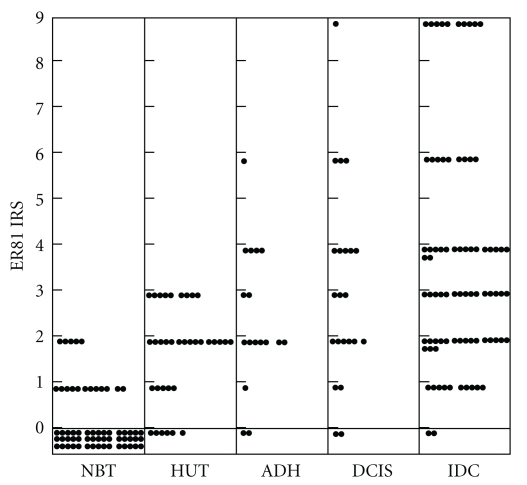
The distribution of ER81 expression in breast cancers and hyperplasia. Each dot represents ER81 IRS of one case.

Formalin-fixed tissue sections from a spectrum of mammary lesions were analyzed for ER81 expression. As shown in Table 1 and Figures 1, 2, and 3, 72.6% normal breast specimens were completely negative for reactivity to ER81, and the rest of the cases reacted very slightly yielding a maximum IRS of 2. Hyperplastic lesions without atypical demonstrated slightly higher levels of ER81 expression than did in nonhyperplasia (*P* < .01) with an average IRS of 1.77 for ER81. In 9 of 35 specimens, hyperplastic epithelium demonstrated IRS  =  3, whereas all of the remaining cases were IRS ≤ 2. Hyperplastic lesions with atypical generally demonstrated slightly higher levels of ER81 expression than did in nonatypical hyperplastic epithelium (*P* < .01) and nonhyperplasia epithelium (*P* < .01) with an average IRS of 2.53 for ER81. 6 of 17 atypical hyperplasia cases were ER81 expression “++” and 1 case was ER81 expression “+++” with IRS  =  6. Ductal carcinoma in situ tissues generally demonstrated slightly higher ER81 expression than did in atypical hyperplasia with an average IRS of 3.18 for ER81, but the difference is not statistically significant (*P* > .05). 4 of 22 (18.2%) ductal carcinoma in situ tissues was ER81 expression “+++” and 8 of 22 (36.4%) cases were ER81 expression “++”. Invasive ductal carcinomas demonstrated enhanced levels of ER81 expression. The expression of ER81 in invasive ductal carcinomas was statistically different from that in ductal carcinomas in situ, atypical ductal hyperplasia tissues, hyperplasia cases of usual type, and normal breast tissues (*P* < .01). The average IRS of invasive ductal carcinomas for ER81 was 3.74, and 19 of 81 cases (23.4%) showed ER81 expression “+++”. The IRS of carcinoma cells varied from 0 to 9 with 51 of 81 cases (62.9%) demonstrating IRS ≥ 3 for ER81 expression.

**Figure 4:**
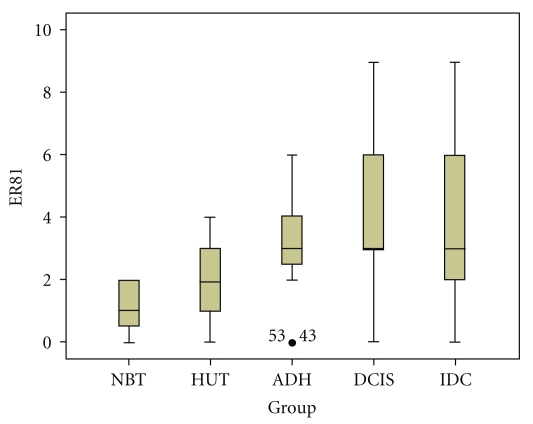
Comparison of ER81 expression in breast cancers combined with DCIS and benign breast hyperplasia. The box plot markings represent median, 25–75th percentile, and the range of all values.

**Figure 5:**
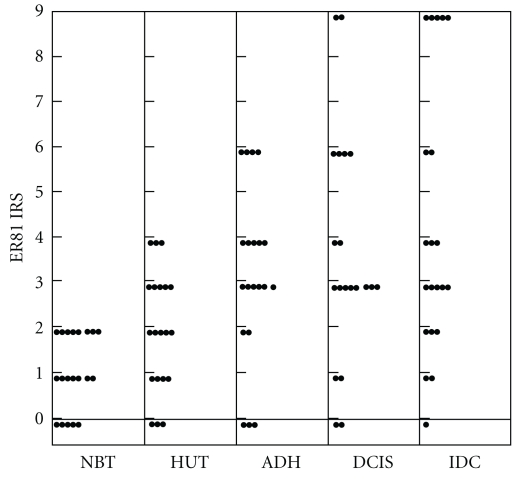
The distribution of ER81 expression in breast cancers combined with DCIS and benign breast hyperplasia. Each dot represents ER81 IRS of one case.

The above data provided lines of evidence that ER81 overexpression happened in the early stage of breast cancer development. In order to confirm the association between ER81 expression and breast tumorigenesis, we employed 20 IDC tissues combined with DCIS, ADH, HUT, and NBT to examine ER81 expression. We considered a threshold IRS set at ≥3 as ER81 positive expression. In this group, ER81 expression was found in 14/20 (70%) IDC. In these 14 cases, ER81 expression was found in 14/14 of adjacent DCIS, 13/14 adjacent ADH, and 8/14 adjacent HUT components. Adjacent normal breast component demonstrated ER81 negative expression. By K-W analysis for the expression level of ER81, we found a statistical significance between NBT and HUT (*P* < .05) and HUT and ADH (*P* < .05), but no statistical significance was found between ADH and DCIS (*P* > .05) or DCIS and IDC (*P* > .05) (Table 2 and Figures 4, 5).

The relationship between ER81 expression and clinical-pathological features such as ER, PR, and Her2 in breast cancer is listed in Table 3. The results revealed that ER81 expression was significantly associated with Her2 amplification and was negatively associated with ER and PR expression. No correlation was found between ER81 expression and patient ages, menopause status, tumor sizes, nodal status, and histological stage (Table 3).

## 4. Discussions

Increasing lines of evidence suggest that breast cancer develop through a multistep model of carcinogenesis, that is, from normal breast epithelia to hyperplasia without atypia, hyperplasia with atypia, ductal carcinoma in situ, to invasive carcinoma [1, 14, 15]. In experiments carried out by DeOme et al. [16], when hyperplastic breast alveolar nodules (HAN), the breast epithelial cells infected by murine mammary tumor virus, were transplanted to cleared mammary fat pads, half of them developed into carcinomas by 13–21 weeks, which happened more frequently than normal breast tissues. In breast biopsies harbouring malignancy, infiltrating carcinoma is often found side-by-side with *in situ *carcinoma and/or benign proliferations. These lesions occasionally show morphological transition and continuity with the invasive carcinoma. Karpas et al. [17] evaluated 645 breast biopsies (226 malignant and 419 benign) and found atypical hyperplasia in 62% of malignant biopsies, but only in 4% of benign biopsies. Similarly, Kern and Brooks [18] found a greater incidence of atypical ductal hyperplasia (ADH) in cancer-bearing breasts. Foote and Stewart [19] found that papillary hyperplasia with atypia occurred five times more frequently in the cancerous breasts. In a similar study, Ryan and Coady [20] found that hyperplasia was four times more common in the cancerous breasts. These histopathological studies data provide convincing evidence that some forms of proliferative lesions are often found in association with invasive cancer and that ADH provides a significantly increased relative risk of subsequent invasive carcinoma. However, little is known about the molecular genetic mechanisms involved in the transformation from hyperplasia to cancer, which is important for the early diagnosis and molecularly targeted therapy of breast cancers [21]. 

ER81 is a downstream gene of *Her2*/Neu, a receptor tyrosine kinase and proto-oncoprotein. And* Her2* is especially associated with breast cancer. Of note, ER81 transcriptional activity is dramatically enhanced upon Her2/Neu overexpression [8, 9]. On the other hand, ER81 can target Her2 and upregulate Her2 expression in breast tumors, suggesting the existence of a feed-forward loop in the upregulation of HER2/Neu [22]. Moreover, ER81 mRNA levels are increased in murine cell lines and tumors overexpressing Her2/Neu and also in many human breast cancer cell lines, suggesting that ER81 may contribute to breast tumorigenesis [10–12]. Shin et al. found that ER81 downregulation suppresses proliferation of *Her2*-positive MDA-MB-231 breast cancer cells in vitro and tumor formation in vivo, proving for the first time the existence of a critical role of ER81 in breast cancer cell physiology [5]. Although transgenic mice overexpressing ER81 in the breast do not develop mammary tumors, ER81 overexpression may prime breast cells to become malignant, for instance upon additional overexpression of Her2/Neu [5].

In this study, we investigated the role of ER81 in breast carcinogenesis by two steps: (1) examining ER81 overexpression in IDC, DCIS, ADH, HUT, and normal breast tissues which represents different stage of breast cancer development. As a result, weak staining was observed in normal breast tissues yielding a maximum IRS of 2. If cells in other groups were considered ER81 positive with IRS ≥ 3, ER81 overexpression was detected in 25.7% (9/35) of HUT, 41.2% (7/17) of ADH, 54.5% (12/22) of pure DCIS, and 63.0% (51/81) of IDC. Although there was ER81 expression in HUT, all ER81 positive tissues were moderate staining. The expression level of ER81 was increased with the progression of the lesion. It is implied that ER81 overexpression are present in the early stage of breast development. (2) Examining ER81 overexpression in breast cancer and the adjacent hyperplasic components (each component represents one stage of breast cancer development) in a single tumor. In this group, ER81 expression was found in 70% (14/20) IDC. In these 14 cases all cases were ER81 positive expression in DCIS, 13 of 14, cases were positively expressed of ER81 in ADH, and 8 of 14 were positive for ER81 in HUT components. A statistical significance was found between NBT and HUT (*P* < .05) and HUT and ADH (*P* < .05), but no statistical significance was found between ADH and DCIS (*P* > .05) or DCIS and IDC (*P* > .05) confirming that ER81 may involve in breast carcinogenesis. 

In addition, we analyzed the relationship between ER81 expression and clinical-pathological features of breast cancer including Her2 amplification and ER, PR expression. The results revealed that ER81 expression was significantly associated with Her2 amplification and was negatively associated with ER and PR expression. No correlation was found between ER81 expression and patient ages, menopause status, tumor sizes, nodal status, and histological stage. In Her2 positive amplification group, the number of ER81 positive expressed cases was more than that in Her2 negative amplification group. As we know, overexpression of ER81 in itself does not lead to breast tumor formation [23], possibly because ER81 requires stimulation in order to become transcriptionally competent and the activation of ER81 is inducible by the *Her2→Ras→Raf→MAP* kinase signaling pathway [9, 24, 25]. These results suggest that* Her2* and ER81 synergize breast carcinogenesis.

Conclusively, ER81 overexpression was present in the early stage of breast development. Together with previous study results, it is suggested that ER81 may play an important role in breast carcinogenesis.

## Figures and Tables

**Table 1:** ER81 expression in breast cancers and hyperplasia (*n*, %).

	−	+	++	+++	Total
NBT	45 (72.6)	17 (27.4)	0 (0.0)	0 (0.0)	62
HUT	6 (17.1)	20 (57.1)	9 (25.8)	0 (0.0)	35
ADH	2 (11.8)	8 (47.1)	6 (35.3)	1 (5.9)	17
DCIS	2 (9.1)	8 (36.4)	8 (36.4)	4 (18.2)	22
IDC	2 (2.5)	28 (34.6)	32 (39.5)	19 (23.4)	81

**Table 2:** ER81 expression in breast cancers combined with DCIS and benign breast hyperplasia (*n*, %).

	−	+	++	+++	Total
NBT	5 (25.0)	15 (75.0)	0 (0.0)	0 (0.0)	20
HUT	4 (20.0)	8 (40.0)	8 (40.0)	0 (0.0)	20
ADH	3 (15.0)	2 (10.0)	11 (55.0)	4 (20.0)	20
DCIS	2 (10.0)	2 (10.0)	10 (50.0)	6 (30.0)	20
IDC	1 (5.0)	5 (25.0)	8 (40.0)	6 (30.0)	20

**Table 3:** Comparison between ER81 expression and clinical data in breast cancers.

	*n*	ER81 negative (%)	ER81 positive (*n*, %)*	*P *(*χ* ^2^)
Age, years				
≤50	48	22 (45.8)	26 (54.2)	.814
>50	33	16 (48.5)	17 (51.5)	
Menopause				
Before	46	22 (47.8)	24 (52.2)	.483
After	35	14 (40.0)	21 (60.0)	
Tumor size, cm				
≤2	15	7 (46.7)	8 (53.3)	.138
2–5	55	28 (50.9)	27 (49.1)	
>5	11	2 (18.2)	9 (81.8)	
Nodal status				
Negative	43	18 (41.9)	25 (58.1)	.463
Positive	38	19 (50.0)	19 (50.0)	
Histological stage				
I	10	6 (60.0)	4 (40.0)	.246
II	54	14 (25.9)	30 (74.1)	
III	17	6 (35.3)	11 (64.7)	
ER				
Negative	25	1 (4.0)	24 (96.0)	.001
Positive	56	22 (39.3)	34 (60.7)	
PR				
Negative	28	3 (10.7)	25 (89.3)	.010
Positive	53	20 (37.7)	33 (62.3)	
HER2 amplification				
Negative	53	19 (35.8)	34 (64.2)	.041
Positive	28	4 (14.3)	24 (85.7)	

*Breast cancer tissues with IRS ≥ 3 were considered as ER81 positive expression.

## Abbreviations

IDC: invasive ductal carcinoma
DCIS: ductal carcinoma in situ
ADH: atypical ductal hyperplasia
HUT: hyperplasia of usual type
NBT: normal breast tissue
IRS: immunoreactive score
ER: estrogen receptor
PR: progesterone receptor.
